# IGF1R controls mechanosignaling in myofibroblasts required for pulmonary alveologenesis

**DOI:** 10.1172/jci.insight.144863

**Published:** 2021-03-22

**Authors:** Hua He, John Snowball, Fei Sun, Cheng-Lun Na, Jeffrey A. Whitsett

**Affiliations:** 1 Division of Pulmonary Biology and; 2 Center for Lung Regenerative Medicine, Perinatal Institute, Cincinnati Children’s Hospital Medical Center, Cincinnati, Ohio, USA.

**Keywords:** Pulmonology, Organogenesis

## Abstract

Ventilation throughout life is dependent on the formation of pulmonary alveoli, which create an extensive surface area in which the close apposition of respiratory epithelium and endothelial cells of the pulmonary microvascular enables efficient gas exchange. Morphogenesis of the alveoli initiates at late gestation in humans and the early postnatal period in the mouse. Alveolar septation is directed by complex signaling interactions among multiple cell types. Here, we demonstrate that IGF1 receptor gene (*Igf1r*) expression by a subset of pulmonary fibroblasts is required for normal alveologenesis in mice. Postnatal deletion of *Igf1r* caused alveolar simplification, disrupting alveolar elastin networks and extracellular matrix without altering myofibroblast differentiation or proliferation. Moreover, loss of *Igf1r* impaired contractile properties of lung myofibroblasts and inhibited myosin light chain (MLC) phosphorylation and mechanotransductive nuclear YAP activity. Activation of p-AKT, p-MLC, and nuclear YAP in myofibroblasts was dependent on *Igf1r*. Pharmacologic activation of AKT enhanced MLC phosphorylation, increased YAP activation, and ameliorated alveolar simplification in vivo. IGF1R controls mechanosignaling in myofibroblasts required for lung alveologenesis.

## Introduction

Formation of the mammalian lung requires precisely orchestrated interactions among a diversity of endothelial, mesenchymal, and epithelial cells regulated by autocrine and paracrine signaling that controls cell proliferation, migration, and production of the extracellular matrix (ECM). Alveologenesis the final phase of lung morphogenesis; in this phase an extensive surface area is created in which endothelial and epithelial cells come into close apposition, which is necessary for efficient gas exchange after birth. Disruption of lung growth and development in the prenatal and perinatal periods results in alveolar simplification, decreasing alveolar surfaces and impairing lung function, causing bronchopulmonary dysplasia (BPD), a common respiratory disorder affecting premature infants (1, 2).

The highly branched structure of the mammalian lung is established by the process of branching morphogenesis, which is substantially completed in the embryonic period of lung development. Extensive tissue remodeling occurs in late gestation during the saccular period of lung development, creating the dilated saccules needed for ventilation after birth (3). Thereafter, alveoli are created by active cell proliferation and tissue remodeling. In the human lung, alveologenesis mainly occurs from approximately 32 weeks of gestation to ages 2–8 (4). In mice, alveoli are formed between postnatal day (P) 4 and 36 (5). Bulk generation of alveoli depends on a process termed “secondary septation,” in which subsets of fibroblasts extend to form alveolar ridges and produce elastin, ECM, and the signaling molecules that regulate mechanical forces guiding alveolar formation (6–8). A subset of mesenchymal cells, PDGFRα^+^ fibroblasts, plays a critical role in the deposition of elastin and the diverse components of the ECM produced during alveologenesis. Deletion of the murine *Pdgfa* or *Pdgfra* genes or ablation of PDGFRα^+^ fibroblasts by diphtheria toxin expression impairs alveologenesis (9–12). Although septal myofibroblasts have long been considered critical for the formation of alveoli, their specific roles in the process of lung formation, homeostasis, or repair remain relatively poorly understood (11). Recent 3D reconstruction analyses of alveolar septal support the concept the myofibroblasts form extended ridges within alveolar walls, which protrude into the alveolar spaces to form an interconnected “fish-net-like” contractile network (6, 13). The role of myofibroblast contraction in the process is supported by recent observations that the inactivation of myosin light chain kinase gene (*Mlck*) inhibited alveologenesis (14).

Myofibroblast functions are strongly influenced by diverse signaling via RTKs, including PDGFRα, RTK-like orphan receptor (ROR), and FGF receptor (FGFR) (10, 15–17). IGF signaling, activated by IGF1 and IGF2, is known to play multiple roles in the regulation of tissue growth and morphogenesis (18–20). Somatic deletion of IGF1 receptor gene (*Igf1r*) disrupted organ growth and lung architecture (21). Recent studies support the potential therapeutic roles of IGF signaling in the prevention of, noting that administration of rhIGF1 and rhIGFBP3 decreases the severity of BPD in preliminary clinical studies in preterm infants (22). However, mechanisms by which IGF1R signals mediate lung growth and morphogenesis or serve to protect the developing lung tissue from injury remain to be clarified. The findings that genetic deletion of *Igf1r* or *Igf1* caused lethal respiratory failure at birth limited the study of the potential role of IGF1R signaling in the postnatal lung (20, 21). In the present work, we produced mice in which the *Igf1r* was conditionally deleted under the control of tamoxifen-induced *Gli1-CreERT2* in a subset of lung fibroblasts (*Igf1r*^Gli1Δ*/*Δ^). Deletion of *Igf1r* in the postnatal period caused alveolar simplification, disrupted ECM deposition, and inhibited the contractile activity of myofibroblasts. IGF1R signaling was required for activation of PI3K/AKT and YAP activity. Pharmacologic activation of AKT restored YAP and p-MLC, improved alveolar simplification in *Igf1r*^Gli1Δ*/*Δ^ mice, and improved alveolar simplification in the developing mouse lung.

## Results

### Postnatal deletion of the Igf1r gene impairs alveolarization.

**Figure 1:**
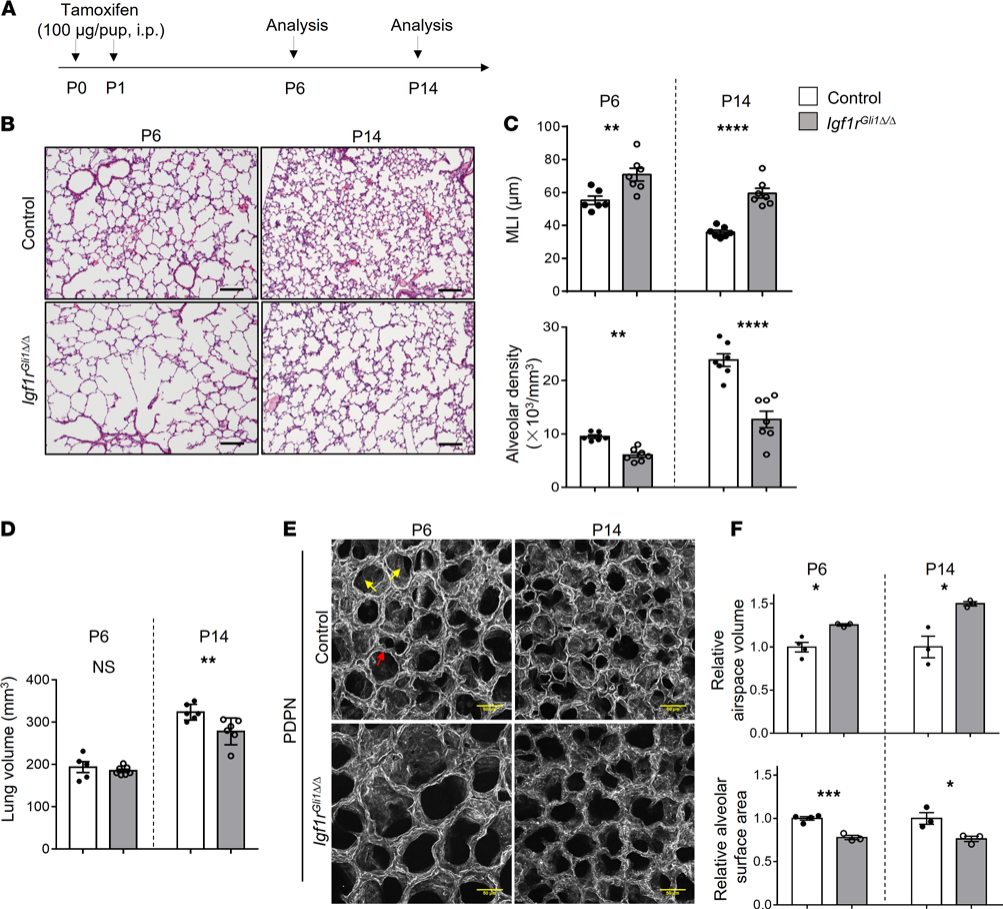
Fibroblast specific inactivation of *Igf1r* causes alveolar simplification. (**A**) Schematic showing the time points of tamoxifen administration and analysis. Tamoxifen was administrated to pups on P0 and P1 via i.p. injection. Lungs were analyzed on P6 and P14. (**B**) Representative H&E staining of control and *Igf1r*^Gli1Δ*/*Δ^ lungs collected on P6 and P14 is shown. Scale bars: 100 μm. (**C**) Mean linear intercept (MLI) and alveolar density on P6; **P < 0.01, *n =* 6 for control and *n =* 7 for *Igf1r* ^Gli1Δ*/*Δ^; and on P14; ****P < 0.0001, *n =* 7 for control or *Igf1r* deleted. (**D**) Lung volume measurement on P6 and P14, **P < 0.01, *n =* 5–7 for each group. (**E**) Reconstruction of 3D confocal images of lungs stained for podoplanin (PDPN) on P6 and P14. Scale bars: 50 μm. Yellow arrows indicate alveolar entrances; red arrow shows secondary septa. (**F**) Quantification of airspace volume and alveolar surface area was measured from surface-rendering images. Airspace volume: P6; *P < 0.05, and P14; *P < 0.05, Alveolar surface area: P6; ***P < 0.001, P14; *P < 0.05, all measurements represent *n =* 3–4 mice per genotype. A 2-tailed Student’s *t* test was used.

**Figure 2:**
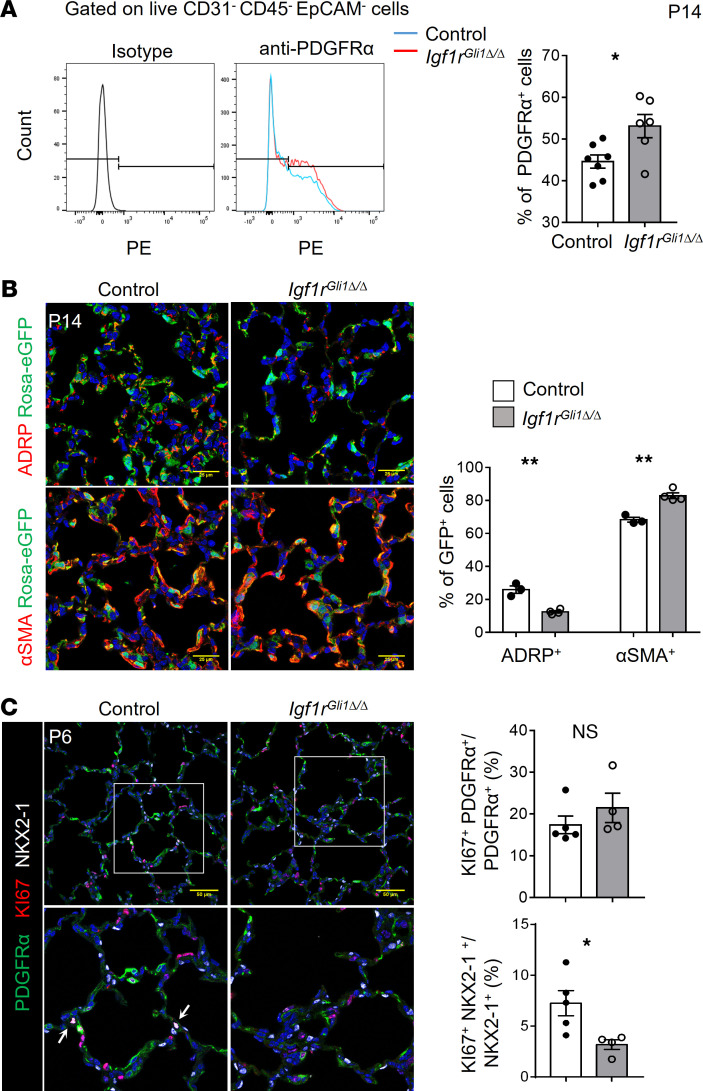
Loss of *Igf1r* does not prevent myofibroblast differentiation and proliferation. (**A**) FACS analysis for PDGFRα^+^ cells in P14 lungs is shown. Cells were stained with CD31, CD45, and EpCAM, and dead cells excluded by DAPI staining. The proportion of PDGFRα^+^ cells was counted in live CD31^–^ CD45^–^ EpCAM^–^ stromal cells. *P < 0.05, *n =* 7 for controls and *n =* 6 for mutants. (**B**) Representative images show immunofluorescence costaining of ADRP or αSMA with GFP on P14 lung sections. Scale bars: 25 μm. Quantification is shown on the right panel. **P < 0.01, *n =* 3 for control and *n =* 4 for mutants. (**C**) Immunofluorescence staining for Pdgfrα, KI67, and NKX2-1 is shown on P6 lung sections. Scale bars: 50 μm. Arrows indicate KI67^+^ NKX2-1^+^ cells. Quantification is shown on the right. For quantification of KI67^+^ PDGFRα^+^/PDGFRα^+^, *P =* 0.33; for quantification of KI67^+^ NKX2-1^+^/NKX2-1^+^, *P < 0.05. All data represent *n =* 4–5 mice of each genotype. A 2-tailed Student’s *t* test was used.

Constitutive deletion of the mouse *Igf1r* gene caused respiratory failure following birth (21). Since the deletion of *Igf1r* with an epithelial-specific *Nkx2-1-Cre* did not alter lung morphogenesis (23), we tested whether its conditional deletion in lung fibroblasts influenced postnatal lung formation. Single-cell RNA sequencing data available on the Lung Gene Expression Analysis (LGEA) website demonstrated that *Igf1r* coexpressed with *Gli1* and *Pdgfra* in a subset of lung fibroblasts on P1 (24) (Supplemental Figure 1A; supplemental material available online with this article; https://doi.org/10.1172/jci.insight.144863DS1). To target these fibroblasts, we employed a *Gli1-CreERT2* mouse line to induce recombination of lox-stop-lox Rosa26-eGFP reporter after treatment with tamoxifen on P0 and P1 and demonstrated that recombination was present primarily in lung fibroblasts and was not detected in epithelial, endothelial, and hematopoietic cells; few airway and vascular smooth muscle cells were targeted (Supplemental Figure 1, B and C). Consistent with previous studies demonstrating that *Gli1-CreERT2* mediated recombination of reporter genes in lung fibroblasts (25, 26), we observed robust recombination of *Rosa26-eGFP* reporter in αSMA-stained myofibroblasts (Supplemental Figure 1D). Recombination was selectively observed in myofibroblasts identified by αSMA staining but was also present in adipocyte differentiation–related protein–stained (ADRP-stained) lipofibroblasts and rarely in PDGFRβ-stained pericytes (Supplemental Figure 1D). To identify the potential role of IGF1R signaling in postnatal lung morphogenesis, we deleted floxed *Igf1r* alleles with *Gli1-CreERT2* by the administration of tamoxifen on P0 and P1 (Figure 1A). We analyzed lung structure on P6 and P14, times corresponding to the early and late stages of septation, respectively. Quantitative PCR (qPCR) analysis demonstrated that *Igf1r* expression was markedly decreased at both developmental stages after treatment with tamoxifen (Supplemental Figure 2A). Likewise, immunofluorescence microscopy demonstrated that IGF1R staining was selectively decreased in Cre-targeted cells, suggesting efficient *Igf1r* deletion (Supplemental Figure 2B). Deletion of *Igf1r* modestly reduced BW in *Igf1r-*deleted mice on P6, weight loss was more significant on P14 (Supplemental Figure 2C). Loss of *Igf1r* caused alveolar simplification as assessed on P6 and P14 (Figure 1B). Morphometric analysis of lung sections showed significantly increased mean linear intercept (MLI) and decreased alveolar density (Figure 1C). Lung volume was significantly decreased on P14 (Figure 1D). 3D confocal immunofluorescence imaging after podoplanin (PDPN) staining demonstrated loss of alveolar septa in *Igf1r*^Gli1Δ*/*Δ^ mice (Figure 1E). Consistent with these morphologic changes, airspace volume was increased and alveolar surface area decreased on P6 and P14 (Figure 1F). Myofibroblasts are known to play important roles in alveologenesis as indicated by lung simplification and disruption of elastin deposition after cell-specific deletion of PDGFRα^+^ cells with diphtheria toxin (9). To assess whether IGF1R signaling was required for PDGFRα^+^ myofibroblast differentiation and proliferation, we introduced a *Rosa26-eGFP* allele into the mutant mice and isolated targeted cells based on GFP expression for RNA-Seq using FACS. Gene Set Enrichment Analysis (GSEA) revealed that the upregulated genes in *Igf1r*^Gli1Δ*/*Δ^ lungs were significantly enriched in signature genes expressed by myofibroblasts as determined by single-cell RNA-Seq analysis from the mouse lung on P3 in the LGEA database (Supplemental Figure 3). FACS analysis revealed a moderate but significantly increased proportion of PDGFRα^+^ cells in the stromal population (Figure 2A). Consistent with this observation, the proportion of lipofibroblasts (ADRP^+^ cells) was decreased and that of αSMA-stained myofibroblasts increased in Rosa26-eGFP–labeled cells of the *Igf1r*^GliΔ*/*Δ^ mice (Figure 2B). The loss of Igf1r activity did not change the proliferation of PDGFRα^+^ cells as determined by quantification of KI67^+^ PDGFRα^+^ cells (Figure 2C). These findings support the concept that myofibroblast proliferation continued in the absence of *Igf1r* expression. In contrast to the relative preservation of myofibroblasts, epithelial cell proliferation, as indicated by NKX2-1 and KI67 colabeled cells, was decreased, whereas the AT1 (HOPX^+^)/AT2 (pro SP-C^+^) ratio of epithelial cells was unchanged (Supplemental Figure 4A), perhaps consistent with decreased alveolar surface area in the simplified lungs. Inhibition of septation in the *Igf1r-*deleted mice was associated with decreased staining of capillary endothelial cells located in alveolar septa (Supplemental Figure 4B).

### Deletion of Igf1r alters the expression of ECM associated genes and disrupts ECM and elastin deposition.

**Figure 3:**
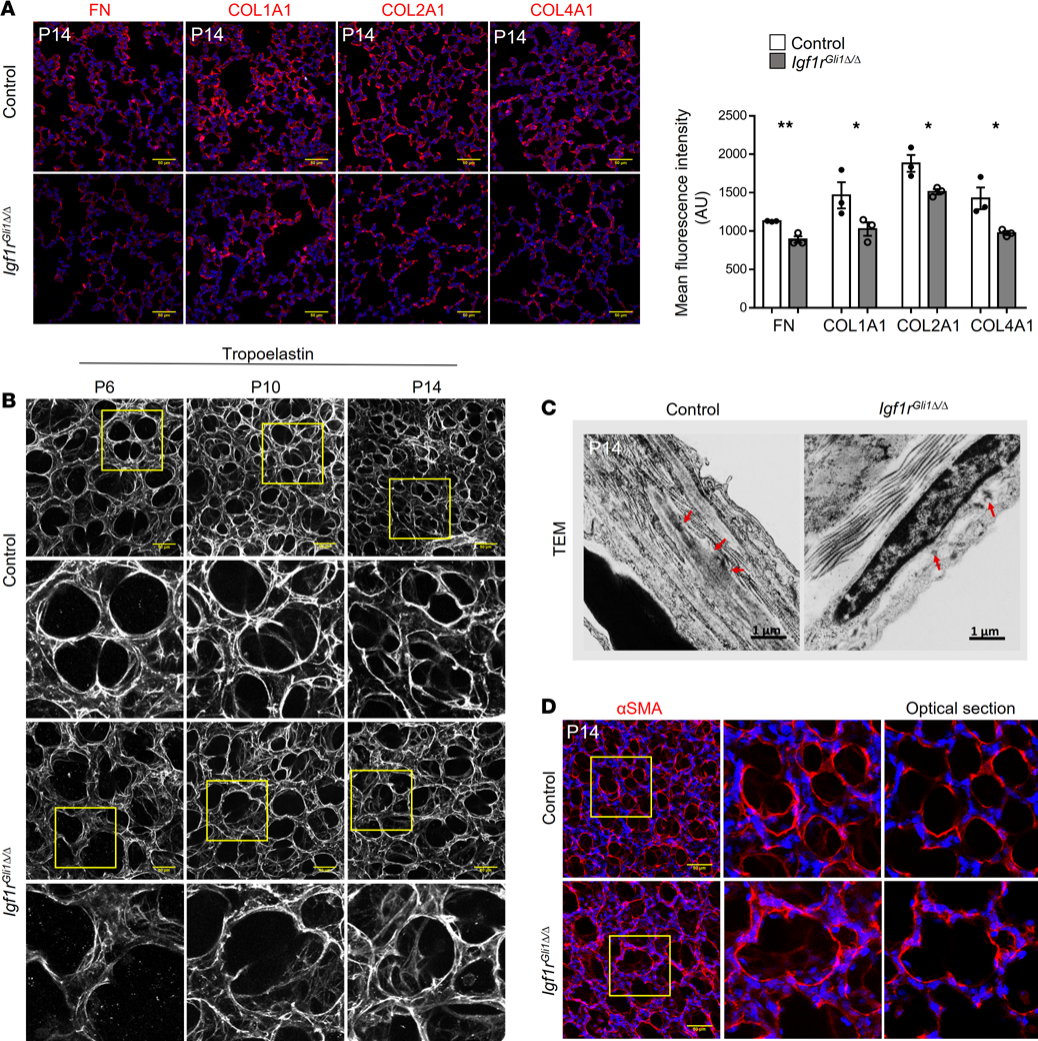
Deletion of *Igf1r* disrupts ECM remodeling, elastin organization and fibroblast morphology. (**A**) Representative images for immunofluorescence staining of fibronectin (FN), COL1A1, COL2A1, and COL4A1 in P14 lung sections. Scale bars: 50 μm. Quantification of mean fluorescence intensity is shown on right panel. *P < 0.05, **P < 0.01, *n =* 3 for each group. A 2-tailed Student’s *t* test was used for each staining. (**B**) 3D reconstruction of confocal images of lungs stained for tropoelastin show disorganized elastin fibers in *Igf1r*^Gli1Δ*/*Δ^ lungs at multiple time points. Tropoelastin staining is well organized in alveolar entrances and septal ridges in control lungs; a less condensed pattern and dispersed fibers in mesenchyme are seen in *Igf1r*^Gli1Δ*/*Δ^ lungs. Scale bars: 50 μm. (**C**) Representative transmission electron microscope (TEM) images show disorganized elastin in *Igf1r*^Gli1Δ*/*Δ^ lungs on P14. Red arrows indicate elastin fibers. (**D**) 3D reconstruction of αSMA immunofluorescence staining of P14 lungs. αSMA staining is condensed in the alveolar entrances and septal ridges in control lungs; diffuse staining is seen in the mesenchyme of *Igf1r*^Gli1Δ*/*Δ^ lungs. Scale bars: 50 μm.

Functional enrichment analysis of RNA-Seq data from GFP-sorted lung cells from *Igf1r*^Gli1Δ*/*Δ^ mice indicated alterations in ECM gene expression associated with the assembly of collagen matrix and contractile fibers (Supplemental Figure 5, A and B). Consistent with these observations, the abundance of ECM components, including fibronectin and collagens, were decreased as assessed by immunofluorescence staining (Figure 3A). Alveologenesis is dependent on the precise deposition of ECM proteins and elastin, which changes dynamically during postnatal development (7, 12, 13). We observed marked defects in the organization of alveolar elastin fibers by 3D confocal imaging, demonstrating that the normal, highly condensed elastin fibers in the alveolar entrance rings and septal ridges were disrupted in the mutant mice, whereas elastin fibers formed abnormal bundles, at alveolar entrances (Figure 3B). Defects in elastin organization were demonstrated by the transmission electron microscope, whereas large elastin bundles were seen in alveolar septa of control mice; only scattered elastin fibers were seen in lungs from *Igf1r*^Gli1Δ*/*Δ^ mice (Figure 3C). Consistent with those findings, expression of genes encoding microfibers, microfibrils, and lysyl oxidases was decreased, all proteins of which are important for elastin fiber and matrix assembly (Supplemental Figure 6). Since myofibroblasts are both contractile and a major source of ECM components, we assessed the organization of myofibroblasts by staining for αSMA. Similar to the patterns of elastin staining, intense αSMA staining formed a fish-net like pattern at alveolar entrances in controls but was diffuse in lung mesenchyme of *Igf1r*^Gli1Δ*/*Δ^ mice (Figure 3D). The shape of GFP-labeled fibroblasts was altered in the *Igf1r* gene-deleted mice, a finding supported by a significant change in “cell shape factor” (Supplemental Figure 7, A and B). Taken together, expression of *Igf1r* in *Gli1* expressing mesenchymal cells plays a critical role in the production and organization of ECM and influences myofibroblast morphology during alveologenesis.

### Igf1r controls myosin-dependent mechanosignaling in lung myofibroblasts.

**Figure 4:**
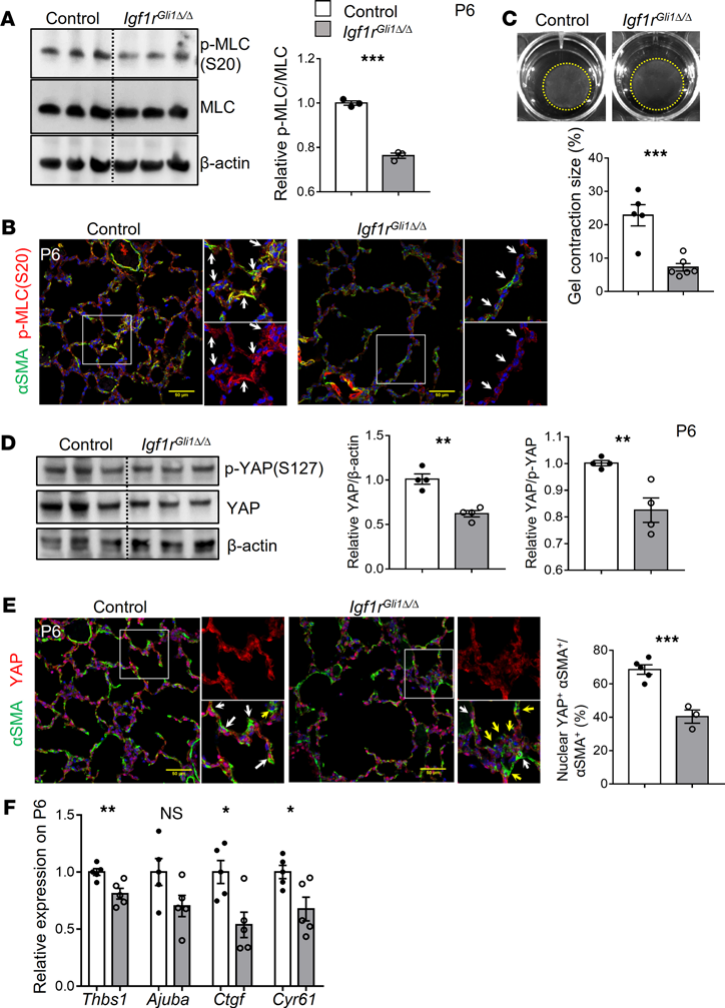
IGF1R controls MLC phosphorylation and YAP activity. (**A**) Western blot analysis of phosphorylated MLC (p-MLC) (S20) and total MLC protein from P6 lung homogenates is shown. Quantification of the mean gray value is shown on the right panel. ***P < 0.001, *n =* 3 for each genotype. (**B**) Immunofluorescence staining for αSMA and p-MLC in P6 lungs. Decreased p-MLC staining of mutant mice is shown. Arrows indicate myofibroblasts. Scale bars: 50 μm. (**C**) Representative images and quantification for the collagen contraction assay show the decreased contractile property of *Igf1r*^Gli1Δ*/*Δ^ myofibroblasts. ***P < 0.001, *n =* 5 for control and *n =* 6 for mutants are shown. (**D**) Western blot analysis of p-YAP (S127) and total YAP protein from P6 lung homogenates. Mean gray value was quantitated on the right panel. Decreased YAP expression and decreased YAP/p-YAP ratio. **P < 0.01, *n =* 4 for control and mutants. (**E**) Immunofluorescence staining for YAP and αSMA on P6 lung sections. Nuclear YAP staining was decreased in mutant mice. Quantification of nuclear YAP^+^ myofibroblasts is shown on the right panel. White arrows indicate myofibroblasts with nuclear YAP; yellow arrows indicate myofibroblasts lacking nuclear YAP. ***P < 0.001, *n =* 5 control, *n =* 3 mutant. Scale bars: 50 μm. (**F**) qPCR analysis of YAP target genes from lung homogenates from P6. **P < 0.01, *P < 0.05, *n =* 5 for each genotype. A 2-tailed Student’s *t* test was used.

Myofibroblast contractility mediated by MLC phosphorylation was recently linked to alveologenesis (14). Because IGF1 signaling is known to mediate the cytoskeletal contractility of multiple cell types (27–30), we tested whether IGF1R signaling influenced the mechanical properties of lung fibroblasts. RNA-Seq data showed that expression of several genes involved in the regulation of cell contraction was altered after deletion of *Igf1r*, including genes encoding regulatory components of G protein–coupled receptor signaling, regulators of intracellular calcium homeostasis and phosphatases (Supplemental Figure 5C). Of interest *Ppp1r12b*, encoding myosin light chain (MLC) phosphatase targeting subunit 2, was increased in *Igf1r*^Gli1Δ*/*Δ^ mice (Supplemental Figure 5C). These findings support the concept that Igf1r signaling may influence MLC phosphorylation controlling myofibroblast contractility. Western blot analysis demonstrated decreased MLC phosphorylation in lung homogenates of the *Igf1r-*deficient mice (Figure 4A). Likewise, immunofluorescence staining demonstrated decreased p-MLC staining in the septal walls of *Igf1r-*targeted mice (Figure 4B). Because stromal cells are able to contract ECM (31), we tested the mechanical properties of myofibroblasts in the *Igf1r-*deficient myofibroblasts. PDGFRα^+^ cells were sorted on P6, and their ability to contract collagen gels in vitro was tested. Contraction of the gel by myofibroblasts from *Igf1r*^Gli1Δ*/*Δ^ mice was significantly impaired (Figure 4C). Since the Hippo/YAP pathway is known to play an important role in mechanical sensing and force production during tissue morphogenesis and repair (32, 33), we tested whether YAP activity was altered in the mutant mice. Western blot of the lung homogenates demonstrated that both YAP expression and the YAP/p-YAP ratio were significantly decreased in *Igf1r*^Gli1Δ*/*Δ^ mice (Figure 4D). Although strong and widespread nuclear YAP staining was observed in myofibroblasts from control lungs, many myofibroblasts in *Igf1r*^Gli1Δ*/*Δ^ lungs showed weak or no nuclear staining (Figure 4E). Quantitative studies demonstrated decreased nuclear staining of YAP in myofibroblasts from the mutant mice (Figure 4E). Consistent with decreased YAP activity, *Thbs1, Ctgf,* and *Cyr61* mRNAs, known transcriptional targets of YAP, were decreased in *Igf1r-*deficient mice on P6 (Figure 4F). Thus, IGF1R signaling regulates mechanosignaling, which involves myosin phosphorylation and YAP activation in lung myofibroblasts during the perinatal period of alveologenesis.

### IGF1R deletion inhibits AKT signaling in fibroblasts

**Figure 5:**
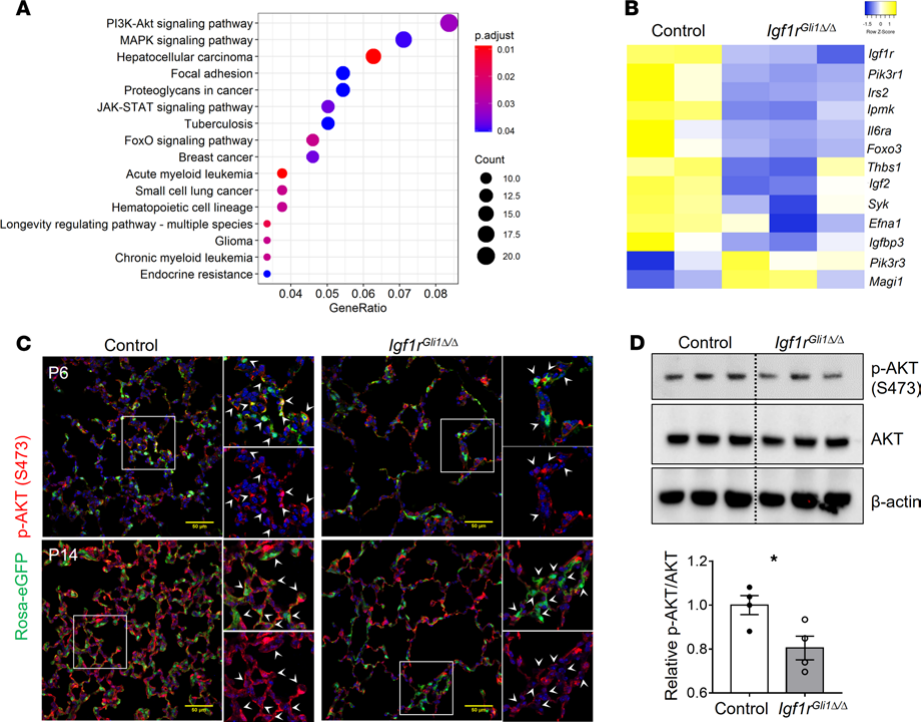
Decreased p-AKT in *Igf1r*-deficient fibroblasts. (**A**) KEGG pathway enrichment analysis identifies major pathways altered. (**B**) Heatmap for selected genes involved in the regulation of PI3K/AKT signaling which were differentially expressed. (**C**) Immunofluorescence staining for GFP and p-AKT (S473) indicates reduction of p-AKT signal in GFP^+^ cells of *Igf1r*^Gli1Δ*/*Δ^ lungs. Arrow heads point to GFP^+^ cells. Scale bars: 50 μm. (**D**) Western blot analyses of p-AKT(S473) and total AKT protein from P6 lung homogenates show decreased AKT phosphorylation in *Igf1r*^Gli1Δ*/*Δ^ lungs, quantification of the integrated density is shown on the bottom panel. *P < 0.05, *n =* 4 each. A 2-tailed Student’s *t* test was used.

Since *Igf1r* is known to regulate a diversity of protein kinases, we sought to identify potential signaling pathways altered in the *Igf1r*^Gli1Δ*/*Δ^ mice. Kyoto Encyclopedia of Genes and Genomics (KEGG) pathway functional enrichment analysis of the mRNAs altered in PDGFRα^+^ fibroblasts identified the PI3K/AKT and MAPK signaling pathways as most influenced by the loss of *Igf1r* (Figure 5, A and B). Confocal immunofluorescence microscopy identified decreased p-AKT staining in Rosa26-eGFP–labeled mesenchymal cells in *Igf1r*^Gli1Δ*/*Δ^ lungs on P6 and P14 (Figure 5C). These findings were supported by decreased p-Akt identified by Western blotting of lung homogenates (Figure 5D). In contrast to the loss of p-AKT, immunofluorescence and Western blot did not reveal changes of p-ERK in lungs of *Igf1r* gene–targeted mice (Supplemental Figure 8, A and B).

### Pharmacologic activation of AKT partially restored alveologenesis, myofibroblast MLC phosphorylation, and YAP activity

**Figure 6:**
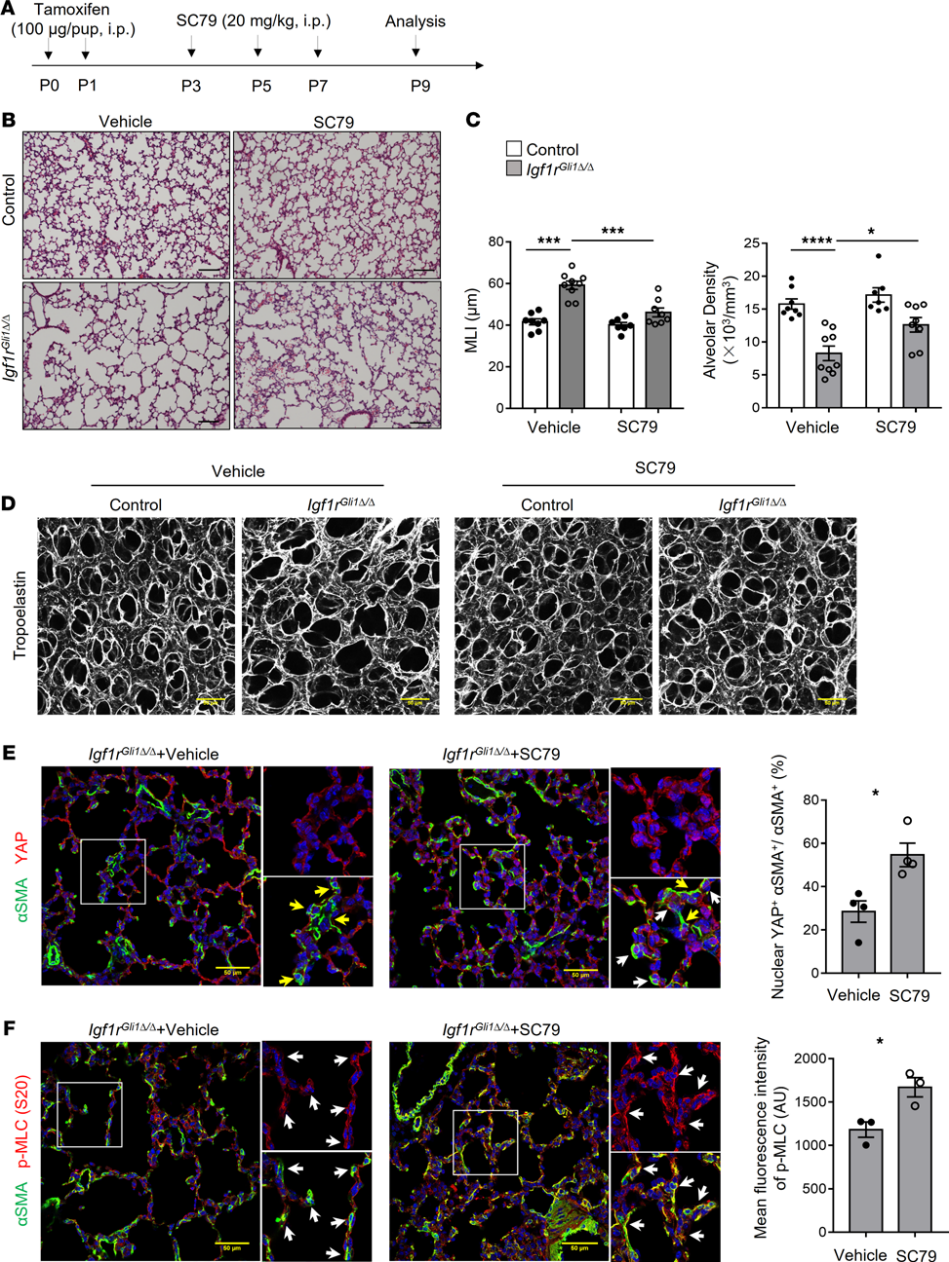
Activation of AKT partially restores alveologenesis, MLC phosphorylation, and myofibroblast YAP activity. (**A**) Schematic shows the time points of tamoxifen and SC79 treatment. Tamoxifen was administrated to pups on P0 and P1 via i.p. injection. SC79 or vehicle was administrated every other day from P3 to P7. Lungs were collected on P9. (**B**) Representative H&E staining of paraffin sections of control and *Igf1r*^Gli1Δ*/*Δ^ lungs treated with vehicle or SC79. Scale bars: 100 μm. (**C**) Mean linear intercept (MLI) and alveolar density are shown. *P < 0.05, ***P < 0.001, ****P < 0.0001, *n =* 8 for control + vehicle, 9 for *Igf1r*^Gli1Δ*/*Δ^ + vehicle, 7 for control + SC79, and 8 for *Igf1r*^Gli1Δ*/*Δ^+ SC79, determined by 1-way ANOVA followed by Tukey’s multiple comparison. (**D**) 3D reconstruction of confocal images of lungs stained for tropoelastin. SC79 partially rescued the disorganized elastin staining in the mutant mice. Scale bars: 50 μm. (**E**) Immunofluorescence staining for YAP and αSMA in vehicle or SC79-treated mutant mice. Quantification of nuclear YAP^+^ in myofibroblasts is shown on the right panel. White arrows indicate nuclear YAP^+^ myofibroblasts. Yellow arrows indicate myofibroblasts lacking nuclear YAP. *P < 0.05, *n =* 4 each. Scale bars: 50 μm. (**F**) Immunofluorescence staining for αSMA and p-MLC (S20) in *Igf1r*^Gli1Δ*/*Δ^ lungs treated with vehicle or SC79. Mean fluorescence intensity of p-MLC is shown on the right panel. Arrows indicate αSMA-stained myofibroblasts. *P < 0.05, *n =* 3 each. Scale bars: 50 μm. A 2-tailed Student’s *t* test was used for **E** and **F**.

Recently, it was reported that AKT signaling integrates the mechanical and cell polarity cues to regulate YAP activity in *Drosophila* and mouse skin models (34). To test whether activation of AKT activity restored alveologenesis, we treated the pups with SC79, a selective activator of AKT (35). SC79 was given at a dose of 20 mg/kg every other day from P3 to P7 (Figure 6A). Mice did not exhibit systemic toxicity as previously reported (35). SC79 activated AKT within 48 hours of treatment in WT animals and induced MLC phosphorylation and YAP as determined by Western blot (Supplemental Figure 9A). AKT activator–treated *Igf1r*^Gli1Δ*/*Δ^ mice exhibited reduced MLI and increased alveolar density compared with mutant treated with vehicle control (Figure 6, B and C), which was accompanied by the improved structure of elastin networks (Figure 6D). Activation of AKT in myofibroblasts from *Igf1r*^Gli1Δ*/*Δ^ mice was confirmed by αSMA and p-AKT costaining (Supplemental Figure 9B). Consistent with the restoration of AKT activity, nuclear YAP and p-MLC were increased in myofibroblasts in *Igf1r*^Gli1Δ*/*Δ^ lungs (Figure 6, E and F). Taken together, present findings demonstrate that *Igf1r* expression in lung mesenchymal cells is required for normal postnatal alveologenesis in a process mediated, at least in part, by the activation of AKT, which regulates mechanosignaling depending on MLC phosphorylation, and YAP to maintain the normal function of lung myofibroblasts during this critical period of lung formation.

## Discussion

Formation of the extensive alveolar surfaces available for gas exchange in the mature lung depends on an orderly process of branching morphogenesis, sacculation, and alveologenesis. Each phase of lung development depends on precise mesenchymal-epithelial interactions that control cell proliferation, migration, and differentiation. Present findings demonstrate that postnatal alveolarization is dependent on IGF1R signaling in a subset of *Gli1* expressing mesenchymal cells. IGF1R was required for the alveolar septal formation and normal production and organization of elastin, ECM, and the contractile activity of myofibroblasts. IGF1R signaling was required for AKT phosphorylation, YAP activation, and MLC phosphorylation in myofibroblasts. Conditional deletion of *Igf1r* in myofibroblasts in the early postnatal period causes alveolar simplification that was ameliorated, in part, by activation of AKT, which restored both nuclear YAP and MLC phosphorylation in septal myofibroblasts. Present findings provide new insights into mechanisms by which growth factor signaling is linked to mechanical force generation required for postnatal lung alveologenesis.

Recent studies support a link between IGF signaling in BPD. Serum IGF1 concentrations are decreased in preterm infants and associated with the severity of BPD (36, 37). Studies in mice (38) and a recent phase 2 clinical trial (22) indicated that treatment with recombinant rhIGF1 and rhIGFBP3 improved pulmonary function, and the incidence of BPD in initial clinical studies. Although we mainly focused on the role of mesenchymal *Igf1r* signaling in the setting of postnatal lung morphogenesis, interruption of perinatal alveolar development also causes lack of reserve lung capacity that may lead to lifelong susceptibility to lung diseases, such as chronic obstructive pulmonary disease and pulmonary hypertension, indicating the potential clinical importance of IGF1R-controlled alveologenesis (39). Importantly, present findings that IGF1R signaling is required for postnatal alveologenesis support the development of potential for therapies related to the activation of IGF1R or signaling through AKT, MLC phosphorylation, or YAP.

The important role of the IGF signaling axis in fetal lung formation is supported by marked growth retardation caused by loss of *Igf1* or *Igf1r*. Deletion of either gene inhibited organ size and caused perinatal lethality in mice (19). Although IGF1 and IGF2 are primarily produced by the liver (40), both ligands and *Igf1r* are expressed in the developing lung, with *Igf1r* broadly expressed in lung mesenchymal and epithelial cells (LGEA database, https://research.cchmc.org/pbge/lunggens/mainportal.html) (24). *Igf1* and *Igf2* are expressed locally, primarily by lung matrix myofibroblasts and smooth muscle cells during the perinatal period of alveologenesis (24). Pharmacologic inhibition of IGF signaling, with a truncated IGF1R, inhibited secondary crest formation in the developing rat lung and inhibited cell proliferation (41). Present findings demonstrated that inhibitory effects on alveolarization are mediated by loss of IGF1R signaling in a subset of *Gli1* expressing lung mesenchymal cells. *Igf1r* was required for the deposition of elastin, ECM and activation of MLC phosphorylation. These findings contrast with the lack of effects of *Igf1r* gene deletion in lung epithelial cells on alveologenesis (23). Although IGF signaling is known to play a role in cell proliferation, deletion of *Igf1r* in the present study did not alter proliferation of PDGFRa expressing myofibroblasts, but did suppress proliferation of alveolar epithelial cells, perhaps consistent with the overall loss of alveolar surface area in the simplified lungs of the *Igf1r*^Gli1Δ*/*Δ^ mice.

Deletion of *Igf1r* caused lung simplification associated with inhibition of p-MLC, a mediator of mechanical force transduction in myofibroblasts and smooth muscle cells. The importance of mechanical force in fetal organogenesis is supported by recent findings that RhoA/ROCK/p-MLC activity was required for branching morphogenesis in the embryonic lung mediated by noncanonical Wnt signaling (42). Loss of p-MLC in the *Igf1r*^Gli1Δ*/*Δ^ mice was associated with decreased YAP protein and decreased canonical YAP target gene expression, e.g., *Cyr61* and *Ctgf*. The decrease in YAP activity seen after deletion of *Igf1r* is similar to findings in *Mlck-*deficient cells (14), with both studies linking YAP activation in myofibroblasts to alveologenesis. The Hippo/YAP pathway regulates diverse cellular activities, including cell proliferation, migration, and organ size, mediated in part by its role in converting mechanical cues to cell signaling activities. YAP and RhoA/ROCK influencing each other’s activity and regulate p-MLC dependent cell mechanics (32, 43, 44). Thus, the present study supports the concept that YAP and MLC interact in a shared pathway to regulate myofibroblast contractility downstream of IGF1R signaling.

Present RNA-Seq analysis of sorted fibroblasts from *Igf1r-*deleted mice detected changes of genes associated with intracellular calcium homeostasis, which is linked to the activation of MLCK (45). The upregulated myofibroblast gene signature in bulk RNA-Seq may reflect the increased myofibroblast cell proportion in mesenchyme, the retained αSMA expression in these cells of *Igf1r*^Gli1Δ*/*Δ^ lungs suggests that IGF1R controls contractility of these cells independently of myofibroblast identity. The preserved αSMA expression in *Mlck* mutant lungs supports this notion (14). Relationships among muscle contraction, calcium homeostasis, AKT, and IGF1R were previously demonstrated in the muscle system (46–48). Consistent with previous findings that AKT activity regulates contractility of lung fibroblasts (49), present data support close relationships among *Igf1r*, myofibroblast contractility, p-AKT, YAP, and alveologenesis. Cell culture studies demonstrated that growth factors, including IGF1, insulin, and VEGF, enhance nuclear YAP localization via AKT signaling (34, 50). Since Hippo kinase activity is dependent on changes of cytoskeleton (51), it is possible that YAP activity in lung myofibroblasts is downstream of MLC phosphorylation. Importantly, although the S127 p-YAP/YAP ratio was decreased in *Igf1r*^Gli1Δ*/*Δ^ lungs (Figure 4D), the decreased level of total protein suggest that other mechanisms might also be involved in regulation of YAP by IGF1R, as there are other protein modifications mediating YAP/TAZ protein stability (52, 53). The finding that pharmacologic activation of AKT restored p-MLC, nuclear YAP and partially restored alveologenesis in *Igf1r-*deficient mice provide support for the role of an integrated mechanosignaling network activated by IGF1R that influences alveolar septation. One of the limitations of the present study is that the systemic administration of AKT activator led to its broad activation in the lung. Although significant Akt activation and its downstream effects were observed in myofibroblasts, Akt activity on other alveolar cells may contribute to the concept of improvement in lung structure after treatment with SC79. In support of this, a recent study demonstrated that AKT activation enhanced migration and repair of airway epithelial cells (54).

In addition to IGF1R, a number of RTKs, e.g., PDGFRα and FGFR3-4, are expressed in subsets of lung mesenchymal cells and are known to play roles in the regulation of myofibroblast activity. Activation of PDGFα and FGFR signaling is known to regulate elastogenesis (10, 17) and both activate cellular processes via PI3K/AKT (55, 56). Recent findings that *Vangl2* regulates AKT activity during alveologenesis and is activated by noncanonical Wnt signaling support the concept that myofibroblast activity is mediated through the orchestration of IGF1R and other RTK signaling and the noncanonical Wnt/PCP pathways (16). How these signals are precisely controlled to direct alveologenesis remains to be determined.

In summary, our data demonstrate that IGF1R signaling in lung fibroblasts plays an important role in the regulation of the contractile properties of myofibroblasts, in part regulated by a pathway, which includes p-MLC, p-AKT, and YAP, providing new mechanistic insight integrating growth factor signaling, mechanical force generation, and alveologenesis in the postnatal lung. Present findings support the concept that activation of IGF1R and AKT signaling represents a potential strategy to maintain or restore defects in alveologenesis in preterm infants at risk for the complications of BPD after birth.

## Methods

### Mice.

Mice carrying *Igf1r*^fl^, *Gli1*-*CreERT2*, and *Rosa26*-*eGFP* have been previously described (57–59) and were interbred to generate conditional knockouts that have an *Igf1r*^fl/fl^ *Gli1*-*CreERT2* genotype. Since *Igf1r^fl/+^ Gli1-CreER* heterozygotes are identical to *Igf1r^fl/fl^* mice, and did not exhibit any phenotype, littermate mice with both genotypes were used as controls. For experiments in which the *Rosa-eGFP* reporter was used, heterozygotes were used as controls. To induce Cre activity, 100 μg tamoxifen (10540-29-1, MP Biomedicals) dissolved in corn oil (5 mg/mL) was injected in both controls and mutants via i.p. on P0 and P1. SC79 or vehicle (5% DMSO + 95% corn oil) was administrated i.p. every other day from P3 to P7 at a dose of 20 mg/kg. All mice were maintained on a mixed background, and both sexes were used in the study.

### Histology, morphometrics, and immunofluorescence analysis.

Lungs were harvested at indicated time points and were gravity inflated with 4% paraformaldehyde in PBS at a 20 cm H_2_O pressure and maintained at 4°C overnight. Tissues were then dehydrated through a series of ethanol and xylene and embedded in paraffin. For morphological analysis, paraffin sections (5 μm thickness) from multiple litters were rehydrated, then stained with H&E. Images were taken on a Nikon Eclipse Ti2 microscope. MLI was calculated as previously described (60). Lung sections were placed under 12 × 10 grid lines, and lines across large blood vessels and major airways were excluded from the study. The number of intercepts between alveolar walls and grid lines was counted, and MLI was calculated using the equation: MLI = total line length/total number of intercepts. Alveolar density was determined by counting alveolar openings in multiple 232.6 × 232.6 μm^2^ frames in the lung parenchyma using paired parallel consecutive histological sections (5 μm thickness) as previously described in detail (61–63); 6–10 random views from multiple lobes and multiple section stages of each lung were used for morphometric quantifications. All analyses were done in FIJI software. Lung volume was measured using the method of water displacement as previously described (61).

For immunofluorescence staining, paraffin sections (5 μm thickness) were rehydrated and placed in Tris-EDTA (pH 9.0) for antigen retrieval using a microwave. Sections were incubated with primary antibodies overnight at 4°C and washed with PBS-Triton X100 (0.3%) 3 times on the following day. Samples were incubated with secondary antibodies at room temperature for 1 hour. After a series of washing with PBS-Triton X100, sections were mounted in the Prolong-Gold mounting media (Thermo Fisher). For protein requiring signal amplification, a Tyramide Signal Amplification (TSA) kit (Perkin Elmer) was used. After washing to remove the unbound primary antibodies, sections were incubated with biotinylated secondary antibodies (Vector Labs), followed by incubation with streptavidin-HRP followed by TSA. A full list of antibody sources and dilutions is shown in Supplemental Table 1. Sections were imaged on Nikon A1R confocal microscopes under identical laser exposures to compare control and mutant lungs. Nuclear staining for KI67 and YAP were counted manually using the “Cell counter” plugin in Fiji software. DAPI circled by αSMA or PDGFRα staining were identified as fibroblast nuclei. For αSMA-stained sections, airway and vascular smooth muscle cells were identified by their anatomical location and were excluded from counting; 5–8 random microscopic views from multiple lobes were acquired and counted for each sample.

3D imaging of tropoelastin, αSMA, and PDPN was performed following a whole-mount staining protocol previously published (64). *Z*-stacks (50 μm) were collected using Nikon A1R confocal microscopes. Images were reconstructed with the maximum intensity projection method using FIJI software. The airspace volume and alveolar surface areas were determined based on the surface rendering of 3D-reconstructed PDPN-stained *Z*-stacks using Imaris software (Bitplane) with default settings. For quantification of “cell shape factor,” 10 μm-thick *Z*-stacks of Rosa26-eGFP signaling were reconstructed with maximum intensity projection, lengths of major axis and minor axis of the best-fitting ellipse for each cell were automatically measured using the Cell profiler 3.0 software (65). Projections of fibroblast were identified by the “IdentifyPrimaryObjects” module: first, a size filter was set at 30–100 pixels to exclude debris, and large clumps and objects touching the border of image were discarded; second, a global 2-classes thresholding with “Otsu” method was applied, threshold smoothing scale was set at 1.9 and threshold correction factor was set at 0.5; third, cell segmentation was done based on “Shape” algorithm, and dividing lines were drawn based on “Intensity” algorithm; fourth, major axis and minor axis lengths of each cell were retrieved using the “MeasureObjectSizeShape” module, and cell shape factor for each cell was calculated. At least 100 cells were analyzed for each sample. The pipeline can be found at GitHub repository: https://github.com/hehua860/pipelines

### Transmission electron microscopy.

Lungs were gravity inflation–fixed with 2% paraformaldehyde, 2% glutaraldehyde, and 0.1% calcium chloride in 0.1 M sodium cacodylate buffer, pH 7.2, followed by immersion fixation with fresh fixative at 4°C overnight. Lung lobes were cut into 1–2 mm blocks and processed for transmission electron microscopy as previously described (66). Images were digitally acquired by an H-7650 transmission electron microscope (Hitachi High Technologies) equipped with a CCD camera (Advanced Microscopy Techniques) at 80 kV.

### FACS and magnetic-activated cell sorting.

Lungs were minced into 1 mm^3^ pieces and incubated with Liberase TM (Roche, 50 μg/mL) and Dnase I (MilliporeSigma, 100 μg/mL) at 37°C for 30 minutes. Tissues were transferred to C-tubes (Miltenyi) and dissociated with a gentle magnetic-activated cell sorting (MACS) dissociator (Miltenyi). Cell suspensions were passed through a 40 μm cell strainer. Cells were subjected to RBC lysis buffer (BioLegend) to remove erythrocytes, and incubated with FcR block (BioLegend, 101319, clone 93, 1:200) on ice for 30 minutes. Cells were then incubated with CD140a (Pdgfrα)-PE (eBioscience, 12-1401-81, clone APA5, 1:200), CD45 -APC-eFluor 780 (eBioscience, 47-0451-82, clone 30-F11, 1:200), CD31-APC (BioLegend, 102509, clone MEC13.3, 1:200), and CD326 (epCAM)-PE-cy7 (eBioscience, 25-5791-80, clone G8.8, 1:200) on ice for 1 hour. Cells were washed with staining buffer, and DAPI was used to exclude dead cells. Data were acquired on a LSR II system (BD Bioscience). FACS sorting of Rosa26-eGFP^+^ cells from single cell suspensions was conducted on a MoFlo XDP system (Beckman Coulter) using a 70 μm nozzle. FlowJo software was used for data analysis (Tree Star Inc.). All flow cytometric data were acquired using equipment maintained by the Research Flow Cytometry Core in the Division of Rheumatology at Cincinnati Children’s Hospital Medical Center. For sorting of PDGFRα^+^ cells, MACS was performed. Single cell suspensions were made as previously described. Cells were incubated with FcR block (Miltenyi, 130-059-901, 1:10) at 4°C for 10 minutes, followed by incubation with CD140a (Pdgfrα) microbeads (Miltenyi, 130-101-502, 1:10) at 4°C for 15 minutes. Cells were washed, resuspended, and passed through a positive selection of LS columns (Miltenyi, 130-042-401) placed on a magnetic field. PDGFRα^+^ cells were then collected in MACS separation buffer (Miltenyi, 130-091-221) for study.

### qPCR and RNA sequencing.

RNA samples were isolated using the RNeasy Mini plus kit (Qiagen) according to the manufacturer’s specification. For qPCR analysis, whole-lung RNA samples were isolated, and first-strand cDNA was synthesized using the iScript Reverse Transcription Supermix (Bio-Rad). Gene expression was normalized to *Gapdh* expression. Taqman Primers are listed in Supplemental Table 2. RNA-Seq was conducted by Genewiz. Sequencing libraries were prepared with the NEBNext Ultra RNA Library Prep Kit (New England Biolabs). Fastq files were processed by Trimgalore and aligned to the mouse genome mm10 using Bowtie2 (67). Raw gene counts were obtained using Bioconductor’s Genomic Alignment, and normalized FPKM values were generated using Cufflinks (68, 69). Differential expression analysis was conducted on raw counts using DeSeq (70). Gene Ontology and KEGG pathway enrichment analysis was performed using clusterProfiler (71) on differentially expressed genes (absolute log_2_ fold change ≥ 0.5, *P <* 0.05 and FPKM>1 in at least one-half of the replicates in one of conditions). Heatmaps were constructed using Heatmapper (http://www.heatmapper.ca/). For GSEA, custom “Matrix fibroblast-1,” “Matrix fibroblast-2,” and “myofibroblast gene set” that consist of the top 100 genes enriched in each cell type were created from the P3 single cell RNA-Seq data in the database (https://research.cchmc.org/pbge/lunggens/mainportal.html). RNA-Seq data are available from the Gene Expression Omnibus database (GSE158451).

### Collagen gel contraction assay.

MACS-sorted PDGFRα^+^ cells from P6 lungs were counted and embedded in rat collagen I (R&D, 1 mg/mL) at 10,0000/well in 24-well plates. The collagen gels were incubated at 37°C for 20 minutes and transferred into 12-well plates with 1 mL DMEM/10% FBS (Gibco) medium. The pictures of gels were taken using ChemiDoc imaging system (Bio-Rad) every 24 hours. Areas of collagen gels from day 5 of culture were measured using FIJI software and compared with those from day 0.

### Western blot.

Lungs were dissected and homogenized in RIPA lysis buffer (Thermo Fisher) supplemented with protease and phosphatase inhibitors (Roche). Denatured protein samples (50 μg) were loaded onto each well of NuPage 4%–12% Bis-Tris gel (Thermo Fisher). Proteins were then transferred onto PVDF membranes and incubated with primary antibodies (listed in Supplemental Table 1) at 4°C overnight. HRP conjugated anti-mouse or anti-rabbit secondary antibodies (Millipore) were used, and peroxidase activity was detected by Immobilon Crescendo Western HRP substrate (Millipore). Western blots were imaged using ChemiDoc imaging system (Bio-Rad). Quantification of integrated intensity was performed using FIJI software.

### Statistics.

Data are presented as mean ± SEM. Unpaired 2-tailed Student’s *t* test was used to determine significance between 2 groups. One-way ANOVA followed by Tukey’s multiple comparison was used to determine significance for more than 2 groups. A *P* value of less than 0.05 was considered significant. GraphPad Prism was used for statistical analysis and graph plotting.

### Study approval.

Mice were housed in pathogen-free conditions according to the protocols approved by the IACUC at Cincinnati Children’s Hospital Research Foundation.

## Author contributions

HH analyzed data and performed functional enrichment analysis of RNA-Seq. JS analyzed RNA-Seq data and edited the manuscript. HH, FS, and CLN performed experiments. JAW interpreted data. HH and JAW designed experiments and cowrote the manuscript.

## Supplementary Material

Supplemental data

## Version 1 02/16/2021

In-Press Preview

## Version 2 03/22/2021

Electronic publication
