# Supplementary material for: IGF1R controls mechanosignaling in myofibroblasts required for pulmonary alveologenesis
Source: JCI Insight. 2021 Mar 22;6(6):e144863. doi: 10.1172/jci.insight.144863 (PMC8026181; doi:10.1172/jci.insight.144863)
Supplement: Supplemental data [file jciinsight-6-144863-s135.pdf]

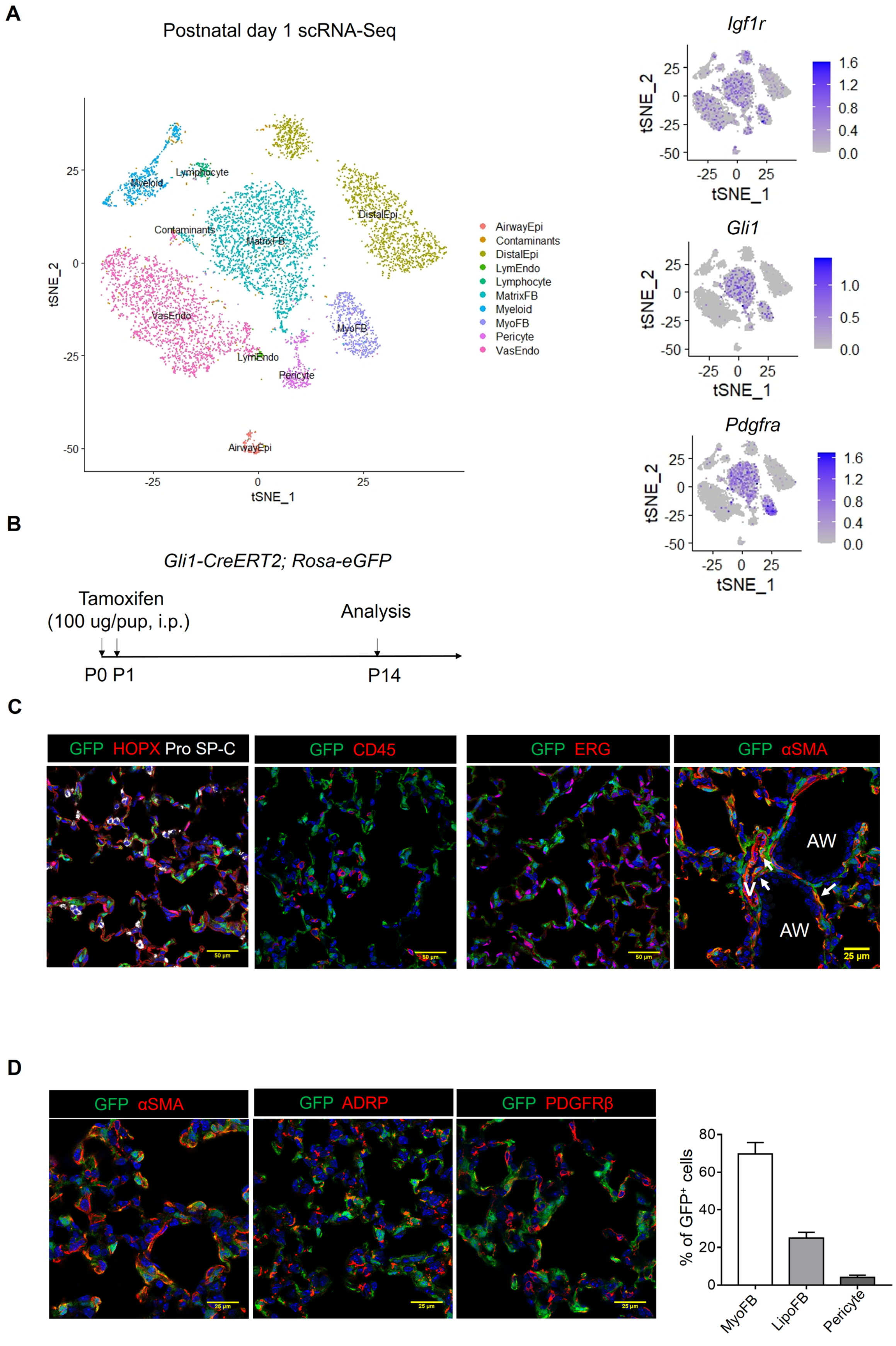

A
Postnatal day 1 scRNA-Seq
tSNE_1
tSNE_2
AirwayEpi
Contaminants
DistalEpi
LymEndo
Lymphocyte
MatrixFB
Myeloid
MyoFB
Pericyte
VasEndo
Igf1r
Gli1
Pdgfra
B
Gli1-CreERT2; Rosa-eGFP
Tamoxifen
(100 ug/pup, i.p.)
Analysis
P0 P1
P14
C
GFP HOPX Pro SP-C
GFP CD45
GFP ERG
GFP αSMA
AW
V
25 μm
D
GFP αSMA
GFP ADRP
GFP PDGFRβ
% of GFP⁺ cells
MyoFB
LipoFB
Pericyte

Supplemental Figure 1. *Igf1r*, *Gli1* and *Pdgfra* expression in Single cell RNA –Seq data from and postnatal *Gli1-CreERT2* activities. (A) Expression of *Igf1r, Gli1* and *Pdgfra* in Single cell RNA –Seq data from postnatal day (P) 1 lungs obtained from LGEA database. (B) Schematic showing the tamoxifen treatment of *Gli1-CreERT2; Rosa-eGFP* mice. (C) Co-staining of Rosa-eGFP reporter with markers for type 2 and type 1 alveolar epithelial cell (Pro SP-C and HOPX), hematopoietic cell (CD45), and endothelial cell (ERG). Note Rosa-eGFP reporter was found in few airway and vascular smooth muscles (arrows). (D) Co-staining of Rosa-eGFP reporter with stromal markers for myofibroblast (αSMA), lipofibroblast (ADRP), and Pericytes (PDGFβ) at P14. MyoFB, myofibroblast; LipoFB, Lipofibroblast. Counting was from 3 mice each for MyoFB or LipoFB, 2 mice for pericytes.

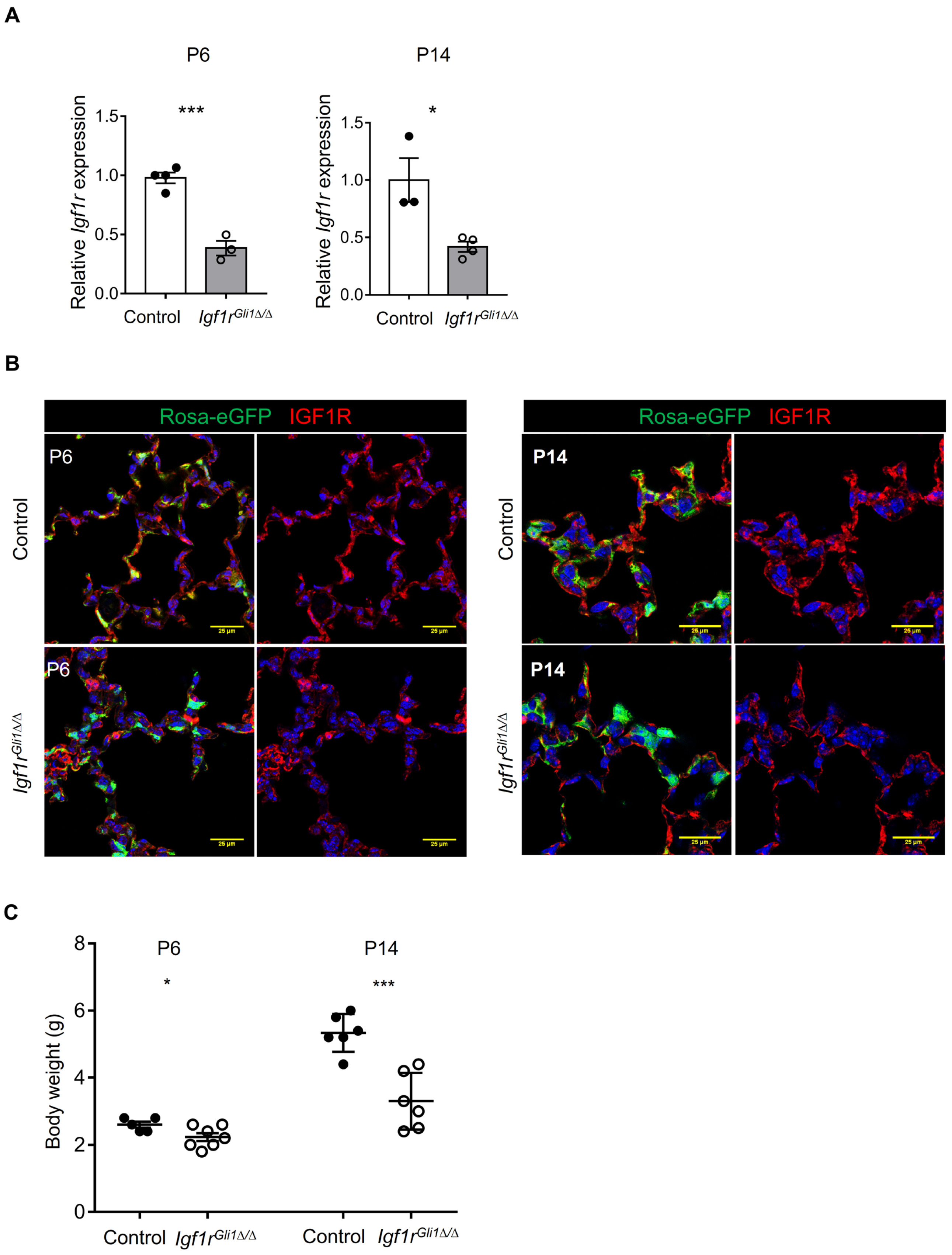

Supplemental Figure 2 Validation of *Igf1r* deletion and mice body weight. (A) QPCR analysis of

Igf1r expression on P6 and P14. **P*<0.05, ****P*<0.001, n=3-4 each group (B) Co-staining of Rosa-eGFP reporter with IGF1R on P6 and P14. (C) Body weight data on P6 and P14 of control and $Igf1r^{Gli1\Delta/\Delta}$ mice, **P*<0.05, ****P*<0.001, n=5-7 each group. A 2-tailed Student's t test was used for each panel.

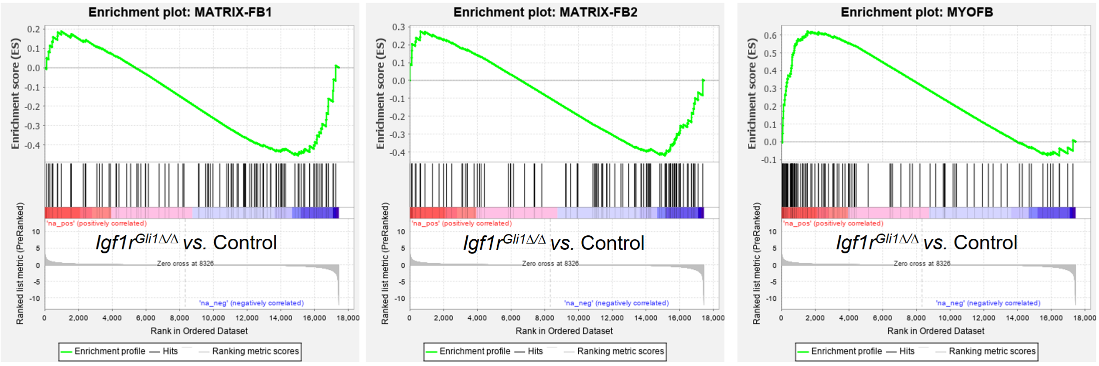

NES=-0.94
q value= 0.57

NES=-1.01
q value= 0.92

NES=1.68
q value= 0.0046

Supplemental Figure 3 Gene Set Enrichment Analysis (GSEA) of altered genes determined by RNA-seq. The transcriptome data from P6 Rosa-eGFP sorted cells from control and *Igf1r*$^{Gli1\Delta/\Delta}$ lungs were analyzed on custom "Matrix fibroblast (FB)-1", "Matrix fibroblast-2" and "Myofibroblast" gene sets that consist of the top 100 genes enriched in each cell type from the P3 single cell RNA-Seq data in LGEA database (https://research.cchmc.org/pbge/lunggens/mainportal.html). There is a significant enrichment of myofibroblast gene set in the upregulated genes in mutant lung fibroblasts.

A

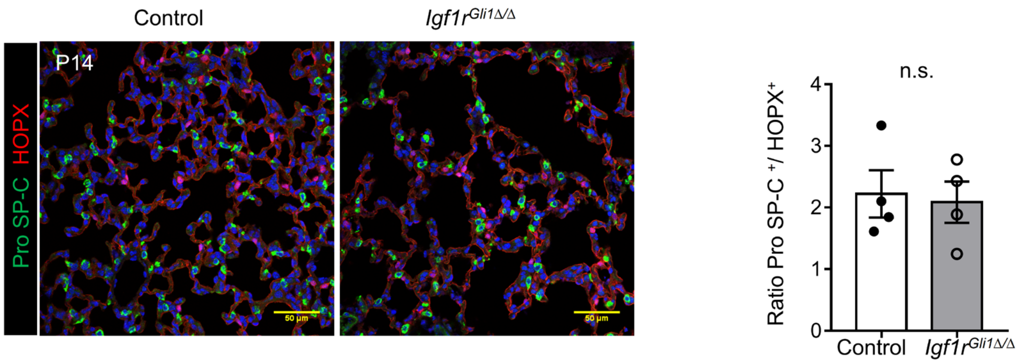

B

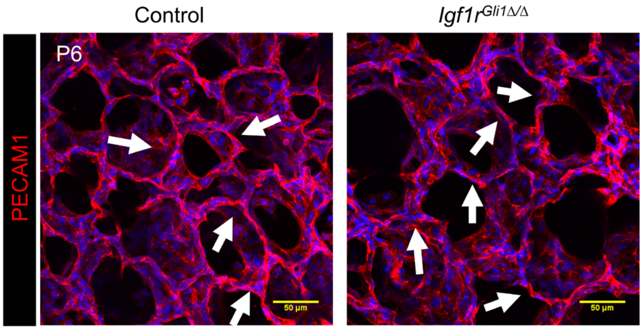

Supplemental Figure 4 Loss of *Igf1r* does not affect alveolar epithelial differentiation but reduces the patterning of endothelial cells at the alveolar septa. (A) P14 control and *Igf1r*$^{Gli1\Delta/\Delta}$ lungs were stained with ProSP-C and HOPX for alveolar type 2 and type 1 cells, respectively. Quantification revealed no changes in their ration between control and *Igf1r*$^{Gli1\Delta/\Delta}$ lungs. n= 4 each, a 2-tailed Student's t test was used. (B) 3D reconstruction of confocal images for Pecam1 staining at P6. PECAM1 staining was decreased at the alveolar septa (arrows) in *Igf1r*$^{Gli1\Delta/\Delta}$ lungs, indicating defective capillary patterning.

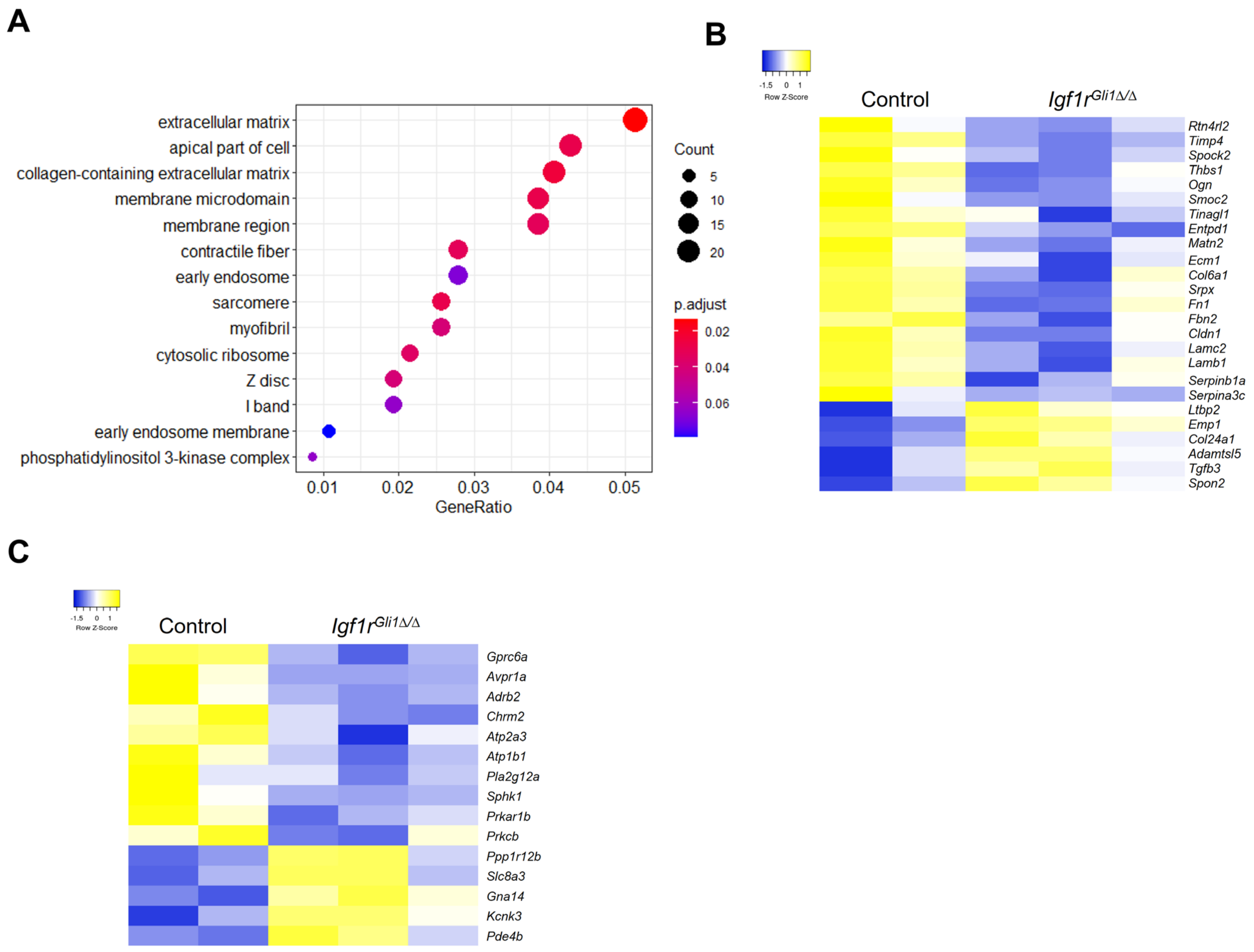

Supplemental Figure5 Loss of *Igf1r* affects the expression of genes associated with extracellular matrix and cell contraction. (A) Enrichment analysis for Gene Ontology Cellular Components of differentially expressed genes obtained from RNA-Seq analysis of FACS sorted *Gli1-CreERT2 Rosa26-eGFP* cells from P6 lungs. (B) Heatmap of selected extracellular matrix genes is shown. (C) Heatmap for differentially expressed genes involved in cell contraction, these include molecules in GPCR signaling, ion channel homeostasis, and phosphatases.

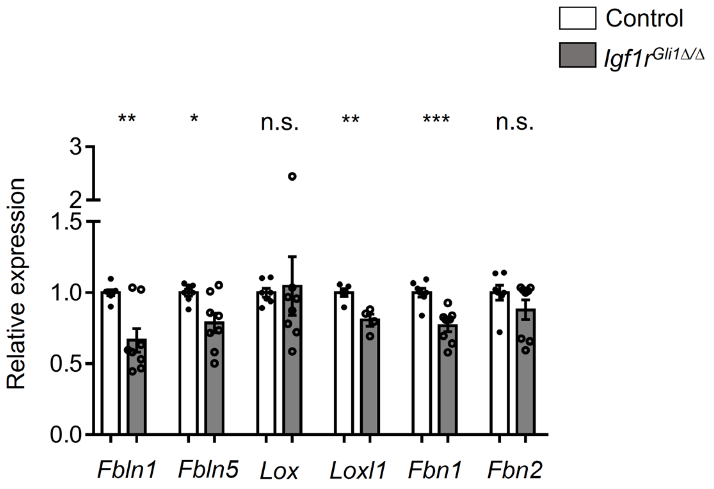

Supplemental Figure 6 Loss of *Igf1r* affects expression of genes associated with elastin assembly. QPCR analysis of elastin genes in control and *Igf1r*$^{Gli1\Delta/\Delta}$ lungs on P6. n=7 for control and 8 for *Igf1r*$^{Gli1\Delta/\Delta}$, *P<0.05, **P<0.01 and ***P<0.001. A 2-tailed Student's t test was used for each assay.

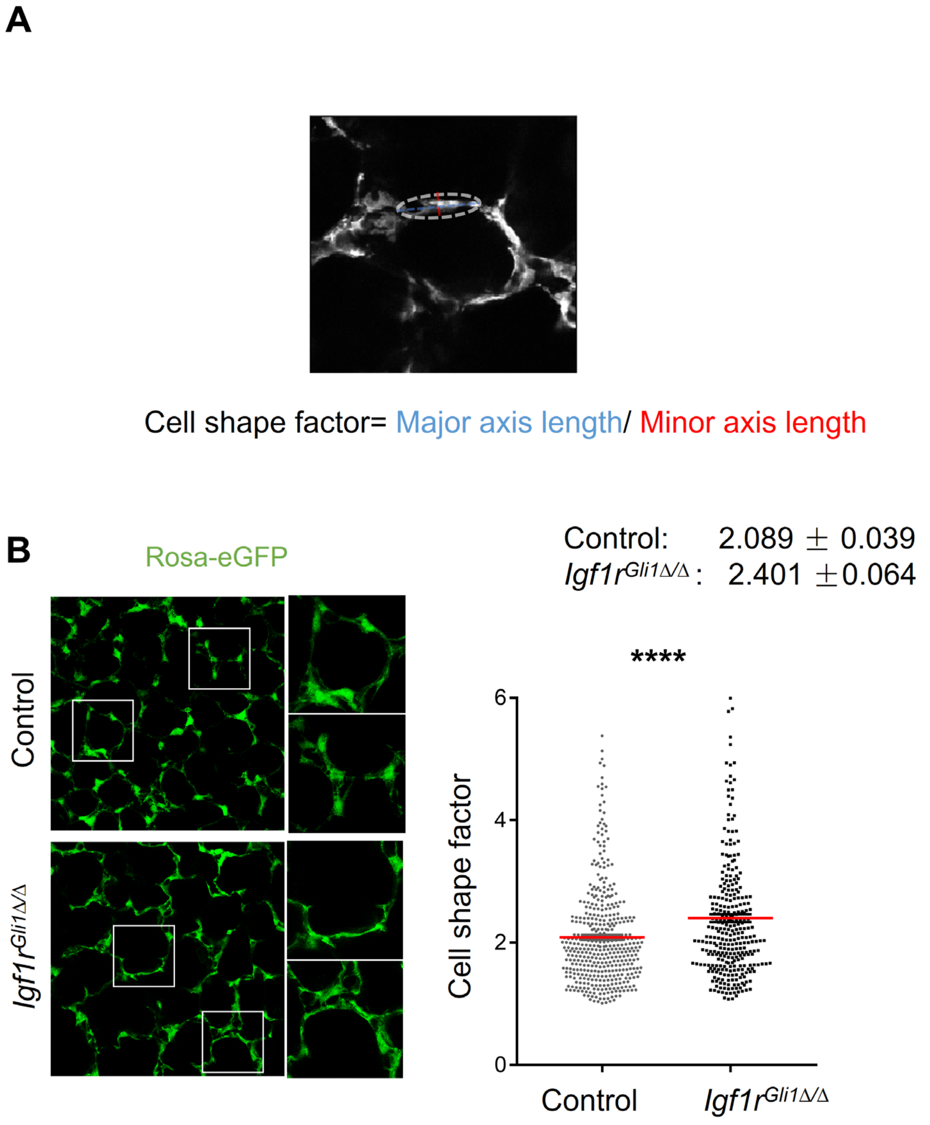

Supplemental Figure 7 Analysis of fibroblast cell shape. (A) Illustration of the definition of "cell shape factor". (B) Maximum intensity projection view of 10 μm-thick Rosa-eGFP confocal images showing the cell shape changes in P14 lungs. The cell shape factor quantification is shown on the right panel. ****P<0.0001, cells for quantification were from 3 controls and 3 mutants. A 2-tailed Student's t test was used.

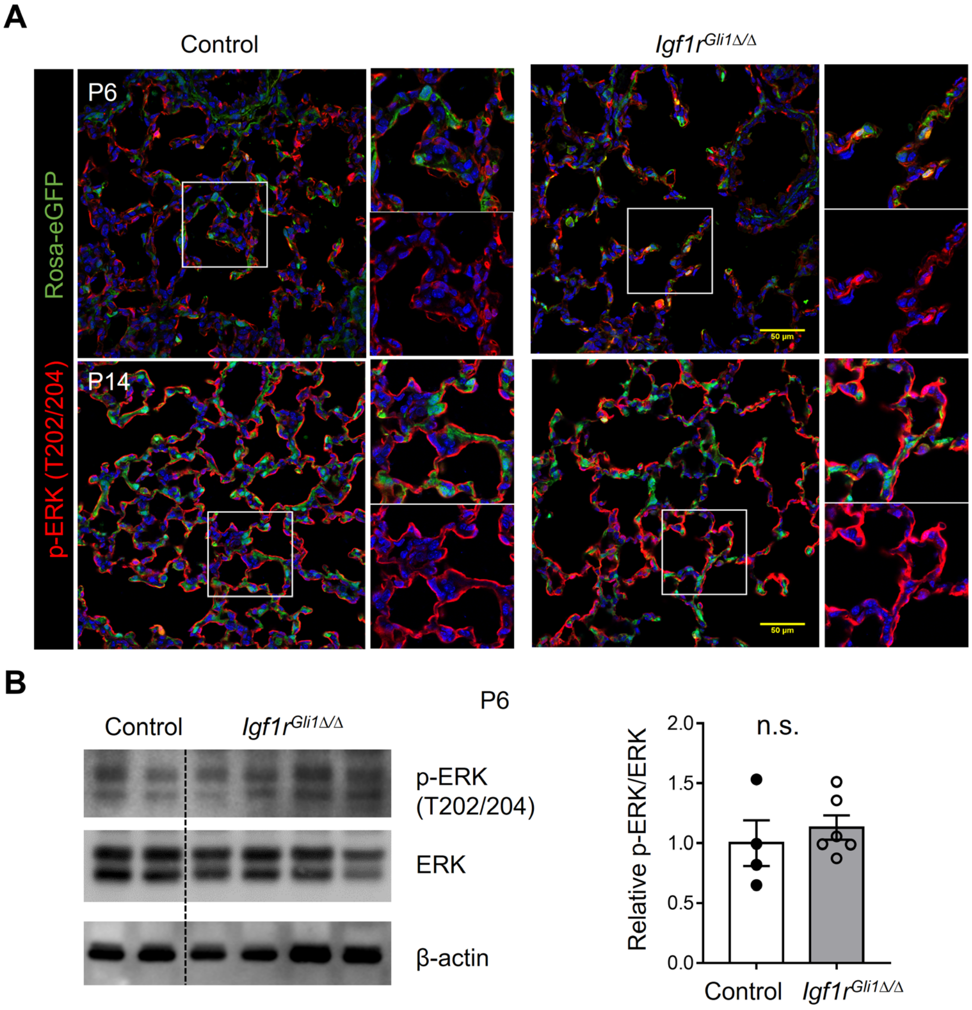

Supplemental Figure 8 Loss of *Igf1r* does not prevent MAPK/ERK activation. (A) Immunostaining for phosphorylated ERK and GFP in P6 and P14 lungs. (B) Western blot analyses of p-ERK and total ERK protein from P6 lung homogenates on P6, quantification was shown on right panel. n= 4 for control, 6 for mutants, a 2-tailed Student's t test was used.

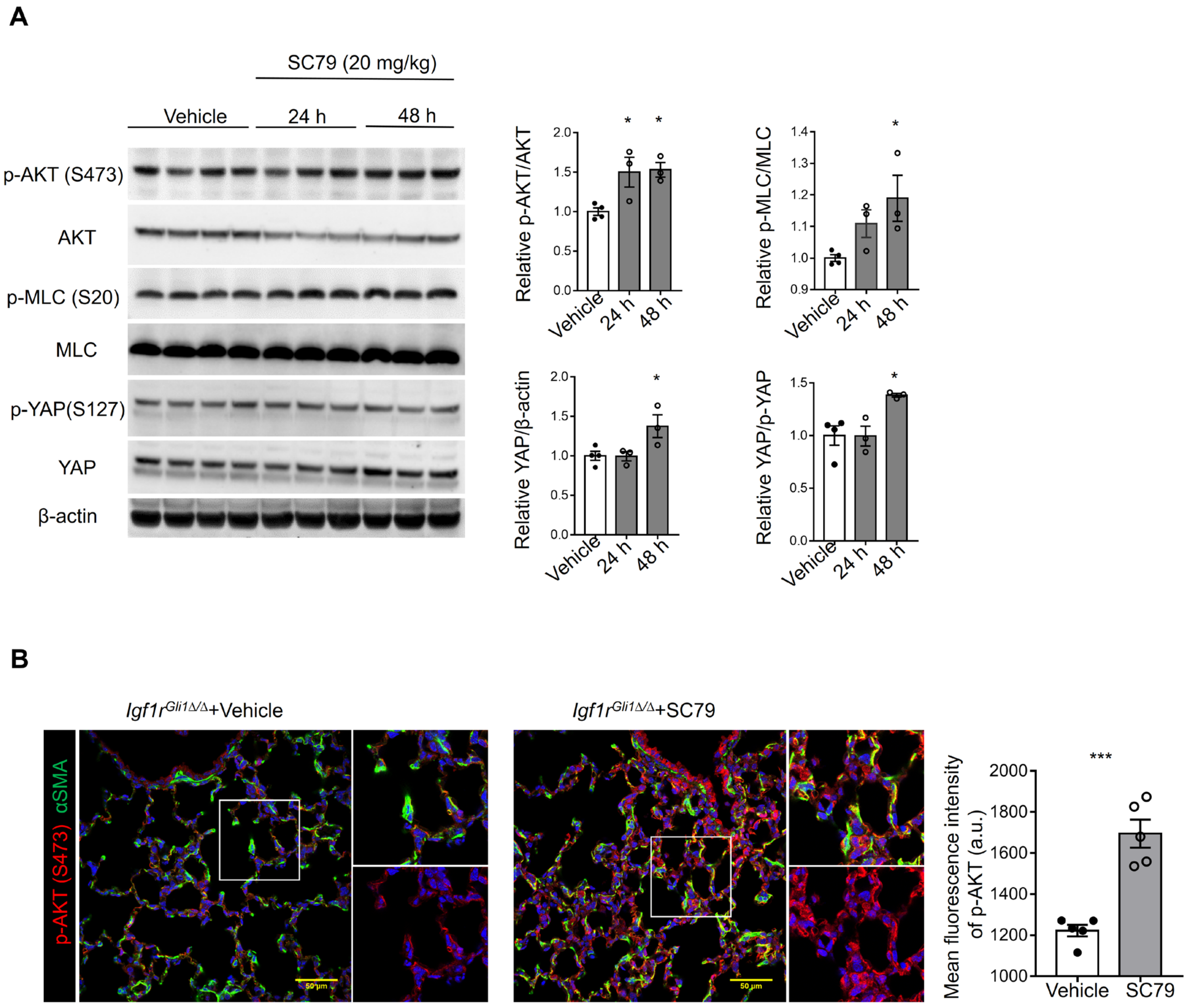

Supplemental Figure 9 Effects of SC79 treatment on wild type and *$Igf1r^{Gli1\Delta/\Delta}$* lungs. (A)Western blot analysis of P5 lung homogenates from wild type mice (C57BL/6) receiving a single i.p. injection of Vehicle or SC79. n=3-4 for each group, *$P<0.05$, determined by one way ANOVA followed by Tukey's multiple comparison. (B) *$Igf1r^{Gli1\Delta/\Delta}$* lungs treated with 3 doses of Vehicle or SC79 as described in Figure 6A were stained for p-AKT and aSMA on P9. Mean fluorescence intensity is shown on the right panel, **$P<0.01$, n=3 for *$Igf1r^{Gli1\Delta/\Delta}$* treated with Vehicle or *$Igf1r^{Gli1\Delta/\Delta}$* treated with SC79, determined by 2-tailed Student's t test.

Supplemental Table 1.
List of primary antibodies used in the study

	Clone No.	Supplier	Catalog No.	Dilution
Podoplanin	8.1.1	Biolegend	127401	1:500 (IF)
ADRP	EPR3713	Abcam	ab108323	1:200 (IF)
αSMA	1A4	Sigma	A5228 or C6198	1:1000 (IF)
PDGFRα	-	R&D systems	AF1062	1:100 (IF)
PDGFRβ	28E1	Cell signaling technology	3169	1:200 (IF)
KI67	B56	BD Pharmingen	556003	1:200 (IF)
NKX2-1	-	Sevenhills	WRAB-1231	1:200 (IF)
Fibronectin	EP5	Santa Cruz	sc-8422	1:200 (IF, TSA)
COL1A1	3G3	Santa Cruz	sc-293182	1:200 (IF, TSA)
COL2A1	B-1	Santa Cruz	sc-518017	1:200 (IF)
COL4A1	COL-94	Santa Cruz	sc-59814	1:200 (IF, TSA)
Tropoelastin	-	Abcam	ab21600	1:500 (IF)
GFP	-	Abcam	ab13970	1:500 (IF)
p-MLC	-	Abcam	ab2480	1:200 (IF), 1:1000 (WB)
MLC	-	Cell signaling technology	3672	1:1000 (WB)
YAP	-	Cell signaling technology	4912	1:200 (IF, TSA), 1:1000 (WB)
p-YAP	-	Cell signaling technology	4911	1:1000 (WB)
AKT	11E7	Cell signaling technology	4685	1:1000 (WB)
ERK	137F5	Cell signaling technology	4695	1:1000 (WB)
p-AKT	D9E	Cell signaling technology	4060	1:200 (IF, TSA), 1:1000 (WB)
p-ERK	D13.14.4E	Cell signaling technology	4370	1:200 (IF, TSA), 1:1000 (WB)
PECAM1	D-11	Santa Cruz	sc-46694	1:200 (IF)
Pro-SP-C	-	Sevenhills	WRAB-9337	1:200 (IF)
HOPX	E-1	Santa Cruz	sc-398703	1:100 (IF)
IGF1R	-	Abcam	ab39675	1:200 (IF, TSA)
ERG	C-1	Santa Cruz	sc-376293	1:200 (IF)
CD45	-	R&D systems	AF114	1:200 (IF)
β-actin	C4	Sevenhills	LMAB-C4	1:5000 (WB)

Supplemental Table 2

List of Taqman primers used in the study

Target	Assay ID
Cyr61	Mm00487498_m1
Gapdh	Mm99999915_g1
Thbs1	Mm00449032_g1
Ajuba	Mm00495049_m1
Ctgf	Mm01192933_g1
Fbln1	Mm00515700_m1
Fbln5	Mm00488601_m1
Fbn1	Mm00514908_m1
Fbn2	Mm00515713_m1
Lox	Mm00495386_m1
Loxl1	Mm01145738_m1
Igf1r	Mm00802831_m1
